# Hepatic Lipid Catabolism via PPARα-Lysosomal Crosstalk

**DOI:** 10.3390/ijms21072391

**Published:** 2020-03-31

**Authors:** Rohit A. Sinha, Sangam Rajak, Brijesh K. Singh, Paul M. Yen

**Affiliations:** 1 Department of Endocrinology, Sanjay Gandhi Postgraduate Institute of Medical Sciences, Lucknow 226014, India; rajaksangam@gmail.com; 2 Program of Cardiovascular and Metabolic Disorders, Duke-NUS Medical School, 8 College Road, Singapore 169587, Singaporepaul.yen@duke-nus.edu.sg (P.M.Y.)

**Keywords:** PPARs, lysosomes, NCoR1, PGC1α, lipophagy, peroxisomes, autophagy, NAFLD

## Abstract

Peroxisome proliferator-activated receptors (PPARs) are ligand-activated transcription factors which belong to the nuclear hormone receptor superfamily. They regulate key aspects of energy metabolism within cells. Recently, PPARα has been implicated in the regulation of autophagy-lysosomal function, which plays a key role in cellular energy metabolism. PPARα transcriptionally upregulates several genes involved in the autophagy-lysosomal degradative pathway that participates in lipolysis of triglycerides within the hepatocytes. Interestingly, a reciprocal regulation of PPARα nuclear action by autophagy-lysosomal activity also exists with implications in lipid metabolism. This review succinctly discusses the unique relationship between PPARα nuclear action and lysosomal activity and explores its impact on hepatic lipid homeostasis under pathological conditions such as non-alcoholic fatty liver disease (NAFLD).

## 1. Introduction

Lipid homeostasis in cells is maintained via a balance of lipid anabolic and lipid catabolic events, which control lipid levels within the hepatic cells [1]. Derangements in this delicate balance of lipid metabolism within the liver cells can lead to metabolic diseases such as non-alcoholic fatty liver disease (NAFLD) and its advance clinical manifestation, non-alcoholic steatohepatitis (NASH) [1]. The incidence of NAFLD has been rapidly increasing worldwide. Targeting hepatic lipid metabolism is currently being investigated as a treatment for NAFLD and its associated conditions such as insulin resistance, cardiovascular disease, and diabetic nephropathy [2]. 

Peroxisome proliferator-activated receptors (PPARs) are key regulators of hepatic lipid metabolism [3,4]. In mammals, three PPAR isoforms have been identified, alpha (α), beta/delta (β/δ), and gamma (γ), which are differentially expressed among various tissues, with PPARα as the predominant isoform in liver [3,4]. The PPARs belong to the nuclear receptor family of ligand-activated transcription factors. The ligands of PPARs include endogenous lipids, such as free fatty acids (FFAs) and eicosanoids. Upon ligand binding, PPARs bind to the PPAR response elements located in promoters of target genes, heterodimerizing with another nuclear receptor, the retinoid X receptor (RXR). Several coactivator and corepressor proteins bind to PPAR/RXR heterodimers to further modulate their transcriptional activity [5]. This PPAR/RXR regulates the expression of genes encoding enzymes or proteins involved in the mitochondrial and peroxisomal β-oxidation, fatty acid (FA) uptake, and lipolysis [6]. Recently, an autophagy-lysosomal mediated lipolysis of triglycerides in liver termed as “lipophagy” was shown to be regulated by PPARα [7]. Additionally, proper lysosomal function was itself determining PPARα transcriptional activity by regulating the stability of its cofactor, peroxisome proliferator-activated receptor gamma coactivator 1-alpha (PGC-1α) [8] and nuclear receptor co-repressor 1 (NCoR1) [9].

In this review, we describe the interplay of this PPARα/lysosomal signaling, which mediates the homeostatic hepatic lipid metabolism. 

## 2. PPARα and Hepatic Lipid Catabolism

PPARα controls the expression of several genes involved in a plethora of lipid metabolic pathways, including microsomal, peroxisomal and mitochondrial β-oxidation, FA binding and activation, FA elongation and desaturation, synthesis and lipolysis, lipoprotein metabolism, gluconeogenesis, and bile acid metabolism [3]. Consistent with its action, PPARα is widely expressed in tissues with high FA oxidation rates, such as heart, liver, and skeletal muscle, and serves as a major regulator of FA homeostasis [10,11]. The human and mouse PPARα genes which share 91% homology are located on chromosome 22 and chromosome 15, respectively [10]. 

PPARα ligands are FA derivatives formed during lipolysis, lipogenesis, or FA catabolism. Animal studies involving genetic disruption of the first rate-limiting peroxisomal β-oxidation enzyme, acyl-CoA oxidase 1 (ACOX1), suggest that its substrates likely are PPARα agonists [12]. Consistently, the deletion of ACOX1 gene in rodents results in increased peroxisome proliferation and elevated PPARα target gene expression [12]. Additionally, eicosanoid derivatives, such as chemoattractant LTB4 and 8(S)-HETE, and murine 8-LOX derivatized from arachidonic acid, also seem to serve as endogenous PPARα agonists [13]. Furthermore, observations suggest that fatty acid synthase (FASN), which is known to be regulated by feeding, is involved in the generation of endogenous PPARα ligands [14]. In addition to its natural ligands, a range of synthetic PPARα agonists, differing in species-specific potencies and efficacies, has been identified. Notably, fibrates such as gemfibrozil, fenofibrate, and ciprofibrate are clinically used in the treatment of lipid disorders such as primary hypertriglyceridemia or mixed dyslipidemia [15]. 

In the absence of specific ligands, PPARα/RXR heterodimers bind to the DNA response elements (PPRE) located in the promoter, enhancer, or intronic region of target genes, and recruit corepressors such as NCoR1, which in turn facilitates histone deacetylases (HDACs) to repress PPARα target gene transcription. However, upon ligand binding nuclear corepressors are released and replaced by coactivators such as PGC1α which, via histone acetylase (HAT) activity, derepress and induce the expression of PPARα target genes involved in hepatic lipid and glucose metabolism. The wide repertoire of genes that is induced in liver after PPARα activation, is suggestive of its central regulatory role in hepatic lipid metabolism [10,16]. These genes include FA transporter, FAT/CD36 and fatty acid-binding protein (L-FABP), and rate-limiting enzymes of peroxisomal β-oxidation, including acyl-CoA oxidase 1 (*ACOX1)* and L-bifunctional enzyme (*EHHADH)*, most pronouncedly in rodents. Additionally, both rodent and primate carnitine palmitoyltransferase I and II (*CPT-I* and *CPT-II)* protein, localized in the outer and inner mitochondrial membrane, respectively, are regulated by PPARα [10,16]. Moreover, PPARα regulates the critical reaction of mitochondrial β-oxidation by directly controlling medium-chain acyl-CoA dehydrogenase (MCAD), long-chain acyl-CoA dehydrogenase (LCAD), very long-chain acyl-CoA dehydrogenase (VLCAD), and mitochondrial 3-hydroxy3-methylglutaryl-CoA synthase (mHMGCoAS) expression levels [10,16]. Studies performed in mice indicate that mechanistic target of rapamycin complex 1 (MTORC1) regulates PPARα activities during the feeding/fasting transition and under pathophysiological conditions. In the fed state, activated MTORC1, through its activation of ribosomal protein S6 kinase beta-2 (S6K2), promotes the nuclear translocation of NCoR1, thereby inhibiting PPARα transcriptional activity. However, the inhibition of MTORC1 and its downstream effector S6K2, during fasting, promotes a cytoplasmic retention of NCoR1 restoring a PPARα mediated increase in genes involved in fat oxidation and ketogenesis [17]. 

## 3. Autophagy and Its Role in Liver Lipid Metabolism

Autophagy is a cellular catabolic mechanism and is a highly conserved recycling process which involves the degradation of cellular constituents in the lysosomes. Although autophagy regulates a number of cell functions, it is primarily involved in maintaining energy balance in liver cells [18]. In the liver, other than maintaining hepatic mitochondrial health in response to energy demand [19], autophagy also helps to provide FAs for mitochondrial oxidation via recycling of hepatic lipid stores [20]. Under lipid loading conditions, hepatocytes in culture accumulate triglycerides (TG) and store them as lipid droplets (LDs) [21]. Intriguingly, both genetic and pharmacological inhibition of autophagy lead to further accumulation of LDs within the hepatocytes, which is associated with defective lipolysis and β-oxidation [21]. However, lipid accumulation is reduced upon autophagy induction. Concurrently, liver-specific deletion of autophagy genes in mice further corroborated these effects on lipid catabolism by displaying increased liver TG and cholesterol levels [21]. 

Therefore, in addition to hepatic lipases such as adipose triglyceride lipase (ATGL and PNPLA2), hepatic lipid stores can be mobilized by a specific subtype of selective autophagy termed as “lipophagy”. Lipophagy targets LDs and catabolizes their components into FFAs and glycerol which are, then, metabolized by the mitochondria [21,22]. The initial stage of lipophagy primarily involves the recognition of LDs by the autophagosomal membrane via the microtubule-associated protein 1 light chain 3 (MAP1LC3), a mammalian homologue of yeast Atg8 and a core component of the phagophore [23]. After subsequent formation of the lipid-laden autophagosomes, these autophagosomes fuse with the lysosomes and the lipid cargo undergoes lipolysis by lysosomal-resident acid lipases [23]. The precise identities of the proteins facilitating these steps of LD recognition are not entirely known, but the polyglutamine protein, Huntingtin, seems to be necessary for lipophagy under stress conditions [24]. Proteins of the Rab family can also play an important role in lipophagy, as many of them have been detected on LDs [25] and some have been associated with autophagy regulation (e.g., Rab7 [26], Rab10 [27], and Rab25 [28]). Interestingly, the cytosolic lipase, ATGL, also facilitates lipophagy suggesting there is a tight co-ordination between cytosolic and lysosomal lipolytic pathways [29,30]. Another lipase, Calcium-independent phospholipase A2-gamma (PNPLA8), also interacts with LC3 to induce lipophagy as part of a SREBP-2-mediated response in a high-fat diet mouse model [31]. Similarly, both PNPLA3 and PNPLA5 mediate lipophagy in human hepatocytes during starvation conditions [31,32]. 

The major lipases involved in lipophagy are the lysosomal acid lipases (LALs) that are capable of catabolizing triacylglycerides, diacylglycerides, cholesteryl esters, and retinyl esters [33,34]. These lipases are mechanistically different from their cytosolic counterparts because of their abilities to function in acidic, rather than neutral environments [35]. The induction of lipophagy is coupled with mitochondrial β-oxidation and treating hepatocytes with lysosomal inhibitors or silencing of autophagy genes leads to increased hepatic triglycerides (TAGs) accumulation and reduced mitochondrial β-oxidation [21,36,37]. The cell signaling pathways involved in regulating lipophagy are similar to general autophagy at the post-translational level and are controlled by the energy- and nutrient-sensing kinases 5′-AMP-activated protein kinase (AMPK) [38,39] and MTOR1 [40], respectively.

## 4. PPARα and Hepatic Autophagy/Lipophagy

Several mechanisms are associated with the regulation of autophagy by PPARs. Notably, PPARγ is known to upregulate the expression of hypoxia-inducible factor 1 (HIF1), and BCL2 interacting protein 3 (BNIP3) to regulate autophagy in breast cancer cells [41]. Additionally, the regulation of AMPK, MTOR1, NEDD4, and uncoupling protein 2 (UCP2) by PPARγ also contributes to autophagy induction in mammalian cells [42,43,44]. However, direct transcriptional regulation of lipophagy has also been shown to be mediated by nuclear hormone receptors such the thyroid hormone receptors (THR) [37], cAMP responsive element binding protein (CREB) [45], farnesoid X receptor (FXR) [7], and PPARα [7]. The function of the liver in the fasted and fed states is strikingly divergent metabolically [1]. In the fed state, the liver switches to an anabolic mode and shuttles nutrients for storage, synthesizing both glycogen and FAs [46]. However, it initiates catabolic functions in the fasted state, including autophagy induction, oxidizing FAs, and synthesizing glucose for utilization by other tissues [46]. FXR and PPARα serve as nutrient sensors which fine tune the transcriptional program under fed and fasted states [47]. Interestingly, in the liver, the increases in PPARα expression and transcriptional activity during starvation are closely related to the induction of autophagy [7]. Furthermore, in the experiments performed in wild type and FXR–/– and PPARα–/– mice treated with or without the FXR and PPARα agonists GW4064 and GW7467 showed that PPARα agonist could induce autophagy in wild type mice liver even in a fed state but not in PPARα–/– mice. Similarly, FXR agonist could also suppress autophagy in a fasted state in an FXR dependent manner. At the transcriptional level, this was associated with opposing effects on expression of a wide range of autophagy-related genes, and genome-wide ChIP-Seq binding studies confirmed that such genes were highly enriched as apparent primary targets of both these nuclear receptors [7]. In addition to the general induction of autophagy, PPARα agonist also specifically induced lipid catabolism through lipophagy [7]. In addition to pharmacologic responses, the induction or repression of autophagy/lipophagy in mice liver was also dependent on the PPARα and FXR expression, respectively. Therefore, these results highlight the existence of a homeostatic role for each receptor in the normal nutrient regulation of the autophagy pathway. 

**Figure 1:**
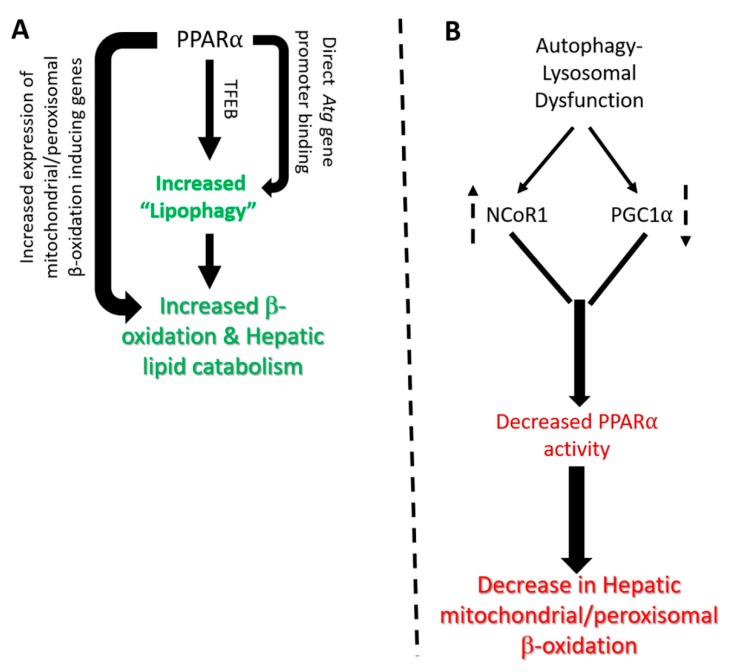
Reciprocal regulation of PPARα and autophagy-lysosomal signaling. (**A**) Induction of PPARα leads to increased transcription of autophagy (*Atg*) genes through either direct binding of PPARα to their promoter or through secondary regulation of TFEB levels. Induction of autophagy genes leads to engulfment of intrahepatic lipid droplets by autophagosomes and their eventual hydrolysis in lysosomal compartment termed as “lipophagy”. The free fatty acids released from lysosomes serve as substrate for mitochondrial β-oxidation further induced by PPARα leading to energy generation; (**B**) Impairment of autophagy-lysosomal activity leads to increased stability of PPARα corepressor NCoR1 as well as decreased stability of PPARα coactivator PGC1α leading to suppression of PPARα transactivation activity and reduced lipid catabolism in liver cells. The dotted up and down arrows denotes increase or decrease in levels.

PPARα directly increases the expression of several autophagy genes by directly binding to their promoters [7] (Figure 1A). Studies focusing on the mechanism of these counteracting effects between PPARα and FXR observed that both PPARα and FXR were capable of binding to the same DR-1 cognate sequence in the promoter of autophagy genes such as Lc3a and Lc3b. [7]. The binding of FXR/RXR heterodimers to this cognate PPRE sequence was associated with FXR agonist-dependent corepressor recruitment, in accordance with the observed transcriptional repression. Therefore, these results indicated that there was a competition between the PPARα/RXR and FXR/RXR heterodimers for the Lc3a and Lc3b promoter sites, with the presence of each agonist increasing the occupancy of its cognate receptor, while decreasing that of the other [7]. Additionally, direct binding to autophagy gene promoter, PPARα, also stimulates the gene expression of transcription factor EB (TFEB), a key regulator of autophagy and lysosome gene transcription to indirectly augment the expression of several autophagy and lysosomal genes involved in lipophagy [48,49] (Figure 1A). Interestingly, upstream energy sensing kinases through posttranslational modification of both PPARα and TFEB via phosphorylation can play a key role in the regulation of lipophagy [50,51]. Therefore, collectively, PPARα coordinates several aspects of lipid catabolism including the degradation of LDs/TGs into free fatty acids by lipophagy, followed by subsequent β-oxidation by peroxisomes and mitochondria (Figure 1A).

## 5. Lysosomes Control PPARα Nuclear Action

Signaling from lysosomes to the nucleus is a relatively new area of signal transduction that is actively being investigated [52]. Therefore, departing from the classical view of lysosomes as merely degradative organelles, studies have now discovered signal transduction pathways which originate from lysosomes and effect nuclear transcriptional machinery [53]. This lysosome-to-nucleus signaling seems to be essential to govern lipid catabolic programs in the liver [54]. It examines the effects of nutrient availability on the transcriptional activity of genes during starvation, feeding, and basal conditions [55]. In this regard, a recent study using a transcriptomic approach has identified the important role(s) of lysosomes in regulating transcription of target genes involved in peroxisomal biogenesis and lipid metabolism [8]. 

Peroxisomes are intimately associated with lipid droplets and mitochondria, and their ability to carry out fatty acid oxidation and lipid synthesis regards them as critical mediators of hepatic lipid metabolism [56]. The key physiological functions of peroxisomes in liver are the β-oxidation of very long chain fatty acids, α-oxidation of branched chain fatty acids, and synthesis of ether-linked phospholipids along with the synthesis of bile acids [57]. The proteins required for the formation of peroxisomes are known as peroxins, and together with the proteins and enzymes involved in peroxisomal lipid oxidation, are under the transcriptional control of PPARα and its coactivator, PGC1α [57]. 

Results by Tan et al. showed that both pharmacological inhibitors of lysosomal activity, as well as genetic knockdown of TFEB significantly suppressed the expression of genes involved in peroxisomal biogenesis and lipid oxidiation [8]. Furthermore, this study revealed that the loss of lysosomal functions leads to protein degradation of PGC1α which leads to decreased expression of several PPARα-regulated peroxisomal genes including PPARα itself [8]. Interestingly, the ectopic rescue via combined overexpression of both PPARα and PGC1α negates the effect of lysosomal inhibition on peroxisomal gene expression [8] (Figure 1B). These findings suggest that there is an important crosstalk between lysosome function and PPARα genes involved in autophagy and peroxisomal activity, and vice versa (Figure 1B). The novel connection between lysosomal function and peroxisomal gene transcription via PGC1α-PPARα nuclear receptor activity raises the possibility that peroxisomal activity can be enhanced by increasing lysosomal activity, especially in disorders linked to peroxisomal defects such as Neimann-Pick disease and X-linked adrenal leukodystrophy [58]. Finally, as PGC1α serves as a common coactivator for several other nuclear receptors involved in hepatic lipid metabolism, it is possible that lysosomal inhibition could modulate other cellular and metabolic pathways mediated by these other nuclear receptors [20]. 

The autophagy-lysosomal pathway also regulates the stability of NCoR1, a transcriptional corepressor associated with PPARα and inhibits its transcriptional activity [9]. Interestingly, the loss of hepatic autophagy in *Atg5*-null mice impairs the production of ketone bodies during fasting by reducing the expression of enzymes involved in β-oxidation through a NCOR1-mediated mechanism [9]. NCoR1 interacts with PPARα to suppress PPARα-mediated transactivation of these target genes. NCoR1 also binds to the autophagosomal resident gamma-aminobutyric acid receptor-associated protein (GABARAP) family of proteins and is degraded by autophagy. Thus, the loss of autophagy leads to an over-accumulation of NCoR1, which then suppresses PPARα activity and results in further impairment of autophagy and lipid oxidation [9] (Figure 1B). Another study further supported the role of autophagy on PPARα action showing that hepatic expression of the class 3 PI3K is essential for metabolic adaptation to starvation in the liver through the control of PPARα transcriptional activity [59]. This study showed that the loss of hepatic expression of class 3 PI3K/Vps15 effected the levels of PPARα ligands, as well as PGC1α and NCoR1 levels [59].

At the mechanistic level, this study showed that both NCoR1 and HDAC3 interacted with LC3 and are degraded through the autophagy-lysosomal pathway under fasting conditions. However, in autophagy deficient *Vps15*-deficient hepatocytes this process is impaired, leading to NCoR1 stabilization and inhibition of hepatic PPARα activity. [59]. Therefore, the authors proposed that the class 3 PI3K/VPS15 exerted a broad transcriptional control in the liver to match autophagic activity with mitochondrial metabolism during fasting, via regulation of nuclear receptor action [59]. Additionally, several autophagy proteins themselves could also regulate NCoR1 corepressor activity by a non-autophagy-mediated mechanism to modify PPARα activity [60,61]. Taken together, these foregoing studies suggest that autophagy-lysosomal activity contributes to PPARα activation during fasting, by promoting degradation of NCoR1 on the one hand, and stabilizing PGC1α on the other hand, to increase the production of lipolysis, β-oxidation, and ketone bodies. (Figure 1B). 

## 6. Implication of PPARα-Lysosomal Crosstalk in NAFLD

NAFLD is a disease spectrum which is one of the most prevalent constituents of the metabolic syndrome in the world [62]. Its more concerning subtype, known as NASH, is accompanied by hepatic inflammation and eventually fibrosis. NASH can further progress to life-threatening cirrhosis and hepatocellular carcinoma, and as such, represents an emerging cause for liver transplantation [63]. It is projected that NAFLD could affect 33.5% of the adult population by 2030, out of which, 27% patients could develop NASH [62]. However, currently, no effective approved therapy other that lifestyle intervention exists for NASH, thereby demanding urgent development and newer treatment modalities for its treatment [64,65]. PPARs have gained attention for their possible anti-NASH action owing to their known anti-steatotic and anti-inflammatory activity in liver [64]. In mice, hepatic PPARα levels increase acutely upon challenge with a high-fat diet (HFD) as an adaptive response [66]; however, in chronic high fat diet (HFD) model, their levels decreased [67]. In humans, hepatic PPARα levels negatively correlated with NASH, and an increase in PPARα expression levels was associated with histological improvement after lifestyle intervention or bariatric surgery [68]. Similarly, PPARα−/− mice exhibited more hepatic triglycerides, oxidative stress, inflammation, and cell death with a significantly higher NAFLD activity score (NAS) when fed HFD as compared with the WT controls fed HFD [4,69]. These findings suggest that PPARα could be a potential therapeutic target for NASH. In this connection, the PPARα agonist, Wy-14643, prevented NASH-induced intrahepatic triglyceride accumulation and liver injury in wild type mice fed a methionine- and choline-deficient diet, but had no effect on PPARα−/− mice fed with the same diet [70]. This study showed that PPARα activation prevents triglyceride accumulation in NASH by increasing fatty acid turnover and catabolism via induction of acyl-CoA oxidase, liver fatty acid binding protein, L-bifunctional enzyme, and peroxisomal ketothiolase gene expression [70]. Similarly, in a rodent G6Pase model of the glycogen storage disease, GSD1a, in which patients developed NASH and cirrhosis, the PPARα mixed agonist, bezafibrate, or selective PPARα agonist, fenefibrate, decreased hepatic triglycerides and increased β-oxidation of fatty acids with a concomitant increase in autophagy [71,72].

Unfortunately, the efficacy of PPARα agonist for the prevention or treatment of NASH found in rodents has not been observed in human trials. Small pilot studies of fibrates in patients with NAFLD did not show any histological improvements in steatosis, inflammation, or fibrosis, nor a reduction in ALT, AST, GGT, bilirubin, or cholesterol, which has led to the discontinuation of its evaluation [73,74]. Yet another study involving 46 patients with NASH demonstrated that four weeks of gemfibrozil treatment resulted in an improvement in serum ALT levels as compared with the non-placebo controls [75]. However, pemafibrate, a novel selective PPAR-α agonist, was shown to ameliorate liver dysfunction in type 2 diabetes patients [76]. Encouragingly, elafibranor a dual PPAR-α/δ agonist, has been shown to resolve NASH after a 52-week treatment indicated by reduced liver enzymes, steatosis, and markers of systemic inflammation and fibrosis [77]. Therefore, general trials with PPARα agonist alone have failed to produce optimal histological improvement of NASH in patients. This apparent discrepancy between the efficacies of PPARα agonist in rodent versus human NAFLD could be due to either a difference in PPARα tissue expression patterns or species-specific differences in PPARα biology [4]. Furthermore, resistance to PPARα activation in human NAFLD could be another possibility.

Both autophagy and lysosomal activity are impaired in human NAFLD and NASH [78,79]. The impairment of autophagy by saturated fatty acids is considered to be due to impaired fusion of autophagosomes with lysosomes [80,81]. Extended exposure to high lipid concentrations alters the lipid composition of membranes or vesicular compartment impairing their fusion [80,81]. Furthermore, high-fat diet also upregulates the expression of vesicular fusion proteins leading to a block in autophagic flux and can explain the altered autophagy after prolonged fatty diets [79]. Attenuation of chaperone-mediated autophagy (CMA) was also observed after lipid challenge [82]. Other reports have demonstrated a decrease in the clearance of autophagosomes attributed to a disturbed acidification of lysosomal compartments or downregulated cathepsin expression as a contributor of autophagy-lysosomal impairment in NAFLD and NASH [83,84,85]. 

Intriguingly, autophagy induction in NAFLD and NASH has been seriously considered as a key treatment regimen [86]. Already, caloric restriction, time-restricted feeding [87], and exercise which are known autophagic stimuli, at least in part, underlie some of their beneficial consequences in liver dysfunction and steatosis [88,89]. Similarly, enhancing autophagy through drugs metformin or the disaccharide trehalose, thyromimetics, green tea and caffeine to enhance lipophagy and beta-oxidation have also shown promising anti-steatogenic effects [36,88,90,91]. In addition, the use of TFEB agonists has recently been the focus of a study based on the demonstration that TFEB overexpression in hepatocytes protects against steatosis and insulin resistance via autophagy in mice fed on a high-fat diet [92]. Consistent with these reports, the activation of TFEB by ezetimibe, an inhibitor of NPC1L1-dependent cholesterol transport, also protects against steatosis and hepatocyte injury [93]. Interestingly, some of these autophagy inducing drugs are already FDA-approved, and ezetimibe has been evaluated in clinical trials for patients with NASH [94], although conclusive results require larger studies. 

Intriguingly, the increased incidence of NAFLD in aged population [95] could also be related to observed reduction in both PPARα [96] and autophagy with aging [97]. Consistent with this, lifestyle modifications such as calorie restriction and exercise which increase autophagy during aging are also known inducers of PPARα and hepatic lipid catabolism [96,97].

Given the role of the autophagy-lysosomal pathway in regulating PPARα levels and transcriptional activity, it is possible that the PPARα activity induced by fibrates could be suboptimal in NAFLD patients due to this accompanying autophagy/lysosomal defect. It is, therefore, intriguing to speculate that induction of autophagy/lysosomal activity in combination with PPARα agonist therapy could yield better results in patients with NAFLD/NASH. In agreement with this notion, autophagy inducers in rodents have been effective in resolving NAFLD and are associated with a corresponding induction of PPARα signaling [36,54]. 

## 7. Conclusions

The recent discoveries relating to mutual regulation autophagy-lysosomal activity and PPARα signaling show that their interactions play important roles in hepatic lipid homeostasis. Further studies are needed to explore the full potential of PPARα agonists as primary or combination therapy with autophagy/lysosomal activators for NAFLD/NASH in humans. Given the importance of these findings that relate to hepatic lipid metabolism, it would be worthwhile to investigate similar crosstalk between the autophagy-lysosomal pathway and other nuclear receptors. 

## Abbreviations

PPARs	Peroxisome proliferator-activated receptors
NAFLD	Non-alcoholic fatty liver disease
HFD	High-fat diet
LAL	Lysosomal acid lipases

## References

[1] Sozio M.S., Liangpunsakul S., Crabb D. (2010). The role of lipid metabolism in the pathogenesis of alcoholic and nonalcoholic hepatic steatosis. Semin. Liver Dis..

[2] Wong V.W. (2018). Current prevention and treatment options for nafld. Adv. Exp. Med. Biol..

[3] Kersten S., Stienstra R. (2017). The role and regulation of the peroxisome proliferator activated receptor alpha in human liver. Biochimie.

[4] Liss K.H., Finck B.N. (2017). Ppars and nonalcoholic fatty liver disease. Biochimie.

[5] Berger J., Moller D.E. (2002). The mechanisms of action of ppars. Annu. Rev. Med..

[6] Dubois V., Eeckhoute J., Lefebvre P., Staels B. (2017). Distinct but complementary contributions of ppar isotypes to energy homeostasis. J. Clin. Investig..

[7] Lee J.M., Wagner M., Xiao R., Kim K.H., Feng D., Lazar M.A., Moore D.D. (2014). Nutrient-sensing nuclear receptors coordinate autophagy. Nature.

[8] Siong Tan H.W., Anjum B., Shen H.M., Ghosh S., Yen P.M., Sinha R.A. (2019). Lysosomal inhibition attenuates peroxisomal gene transcription via suppression of ppara and ppargc1a levels. Autophagy.

[9] Saito T., Kuma A., Sugiura Y., Ichimura Y., Obata M., Kitamura H., Okuda S., Lee H.C., Ikeda K., Kanegae Y. (2019). Autophagy regulates lipid metabolism through selective turnover of ncor1. Nat. Commun..

[10] Bougarne N., Weyers B., Desmet S.J., Deckers J., Ray D.W., Staels B., De Bosscher K. (2018). Molecular actions of pparalpha in lipid metabolism and inflammation. Endocr. Rev..

[11] Feige J.N., Gelman L., Michalik L., Desvergne B., Wahli W. (2006). From molecular action to physiological outputs: Peroxisome proliferator-activated receptors are nuclear receptors at the crossroads of key cellular functions. Prog. Lipid Res..

[12] Fan C.Y., Pan J., Usuda N., Yeldandi A.V., Rao M.S., Reddy J.K. (1998). Steatohepatitis, spontaneous peroxisome proliferation and liver tumors in mice lacking peroxisomal fatty acyl-coa oxidase. Implications for peroxisome proliferator-activated receptor alpha natural ligand metabolism. J. Biol. Chem..

[13] Yu K., Bayona W., Kallen C.B., Harding H.P., Ravera C.P., McMahon G., Brown M., Lazar M.A. (1995). Differential activation of peroxisome proliferator-activated receptors by eicosanoids. J. Biol. Chem..

[14] Chakravarthy M.V., Lodhi I.J., Yin L., Malapaka R.R., Xu H.E., Turk J., Semenkovich C.F. (2009). Identification of a physiologically relevant endogenous ligand for pparalpha in liver. Cell.

[15] Staels B., Maes M., Zambon A. (2008). Fibrates and future pparalpha agonists in the treatment of cardiovascular disease. Nat. Clin. Pract. Cardiovasc. Med..

[16] Pawlak M., Lefebvre P., Staels B. (2015). Molecular mechanism of pparalpha action and its impact on lipid metabolism, inflammation and fibrosis in non-alcoholic fatty liver disease. J. Hepatol..

[17] Sengupta S., Peterson T.R., Laplante M., Oh S., Sabatini D.M. (2010). Mtorc1 controls fasting-induced ketogenesis and its modulation by ageing. Nature.

[18] Singh R., Cuervo A.M. (2011). Autophagy in the cellular energetic balance. Cell Metab..

[19] Sinha R.A., Singh B.K., Zhou J., Wu Y., Farah B.L., Ohba K., Lesmana R., Gooding J., Bay B.H., Yen P.M. (2015). Thyroid hormone induction of mitochondrial activity is coupled to mitophagy via ros-ampk-ulk1 signaling. Autophagy.

[20] Sinha R.A., Singh B.K., Yen P.M. (2017). Reciprocal crosstalk between autophagic and endocrine signaling in metabolic homeostasis. Endocr. Rev..

[21] Singh R., Kaushik S., Wang Y., Xiang Y., Novak I., Komatsu M., Tanaka K., Cuervo A.M., Czaja M.J. (2009). Autophagy regulates lipid metabolism. Nature.

[22] Kounakis K., Chaniotakis M., Markaki M., Tavernarakis N. (2019). Emerging roles of lipophagy in health and disease. Front. Cell Dev. Biol..

[23] Maus M., Cuk M., Patel B., Lian J., Ouimet M., Kaufmann U., Yang J., Horvath R., Hornig-Do H.T., Chrzanowska-Lightowlers Z.M. (2017). Store-operated Ca(2+) entry controls induction of lipolysis and the transcriptional reprogramming to lipid metabolism. Cell Metab..

[24] Rui Y.N., Xu Z., Patel B., Chen Z., Chen D., Tito A., David G., Sun Y., Stimming E.F., Bellen H.J. (2015). Huntingtin functions as a scaffold for selective macroautophagy. Nat Cell Biol.

[25] Kiss R.S., Nilsson T. (2014). Rab proteins implicated in lipid storage and mobilization. J. Biomed. Res..

[26] Schroeder B., Schulze R.J., Weller S.G., Sletten A.C., Casey C.A., McNiven M.A. (2015). The small gtpase rab7 as a central regulator of hepatocellular lipophagy. Hepatology.

[27] Li Z., Schulze R.J., Weller S.G., Krueger E.W., Schott M.B., Zhang X., Casey C.A., Liu J., Stockli J., James D.E. (2016). A novel rab10-ehbp1-ehd2 complex essential for the autophagic engulfment of lipid droplets. Sci. Adv..

[28] Zhang Z., Zhao S., Yao Z., Wang L., Shao J., Chen A., Zhang F., Zheng S. (2017). Autophagy regulates turnover of lipid droplets via ros-dependent rab25 activation in hepatic stellate cell. Redox Biol..

[29] Martinez-Lopez N., Garcia-Macia M., Sahu S., Athonvarangkul D., Liebling E., Merlo P., Cecconi F., Schwartz G.J., Singh R. (2016). Autophagy in the cns and periphery coordinate lipophagy and lipolysis in the brown adipose tissue and liver. Cell Metab..

[30] Sathyanarayan A., Mashek M.T., Mashek D.G. (2017). Atgl promotes autophagy/lipophagy via sirt1 to control hepatic lipid droplet catabolism. Cell Rep..

[31] Negoita F., Blomdahl J., Wasserstrom S., Winberg M.E., Osmark P., Larsson S., Stenkula K.G., Ekstedt M., Kechagias S., Holm C. (2019). Pnpla3 variant m148 causes resistance to starvation-mediated lipid droplet autophagy in human hepatocytes. J. Cell. Biochem..

[32] Dupont N., Chauhan S., Arko-Mensah J., Castillo E.F., Masedunskas A., Weigert R., Robenek H., Proikas-Cezanne T., Deretic V. (2014). Neutral lipid stores and lipase pnpla5 contribute to autophagosome biogenesis. Curr. Biol..

[33] Warner T.G., Dambach L.M., Shin J.H., O’Brien J.S. (1981). Purification of the lysosomal acid lipase from human liver and its role in lysosomal lipid hydrolysis. J. Biol. Chem..

[34] Schulze R.J., Sathyanarayan A., Mashek D.G. (2017). Breaking fat: The regulation and mechanisms of lipophagy. Biochim. Biophys. Acta Mol. Cell Biol. Lipids.

[35] Zechner R., Madeo F., Kratky D. (2017). Cytosolic lipolysis and lipophagy: Two sides of the same coin. Nat. Rev. Mol. Cell Biol..

[36] Sinha R.A., Farah B.L., Singh B.K., Siddique M.M., Li Y., Wu Y., Ilkayeva O.R., Gooding J., Ching J., Zhou J. (2014). Caffeine stimulates hepatic lipid metabolism by the autophagy-lysosomal pathway in mice. Hepatology.

[37] Sinha R.A., You S.H., Zhou J., Siddique M.M., Bay B.H., Zhu X., Privalsky M.L., Cheng S.Y., Stevens R.D., Summers S.A. (2012). Thyroid hormone stimulates hepatic lipid catabolism via activation of autophagy. J. Clin. Investig..

[38] Li Y., Yang P., Zhao L., Chen Y., Zhang X., Zeng S., Wei L., Varghese Z., Moorhead J.F., Chen Y. (2019). Cd36 plays a negative role in the regulation of lipophagy in hepatocytes through an ampk-dependent pathway. J. Lipid Res..

[39] Seo A.Y., Lau P.W., Feliciano D., Sengupta P., Gros M.A.L., Cinquin B., Larabell C.A., Lippincott-Schwartz J. (2017). Ampk and vacuole-associated atg14p orchestrate mu-lipophagy for energy production and long-term survival under glucose starvation. Elife.

[40] Zhang H., Yan S., Khambu B., Ma F., Li Y., Chen X., Martina J.A., Puertollano R., Li Y., Chalasani N. (2018). Dynamic mtorc1-tfeb feedback signaling regulates hepatic autophagy, steatosis and liver injury in long-term nutrient oversupply. Autophagy.

[41] Zhou J., Zhang W., Liang B., Casimiro M.C., Whitaker-Menezes D., Wang M., Lisanti M.P., Lanza-Jacoby S., Pestell R.G., Wang C. (2009). Ppargamma activation induces autophagy in breast cancer cells. Int. J. Biochem. Cell Biol..

[42] Iannucci L.F., Sun J., Singh B.K., Zhou J., Kaddai V.A., Lanni A., Yen P.M., Sinha R.A. (2016). Short chain fatty acids induce ucp2-mediated autophagy in hepatic cells. Biochem. Biophy. Res. Commun..

[43] Xi X., Zou C., Ye Z., Huang Y., Chen T., Hu H. (2019). Pioglitazone protects tubular cells against hypoxia/reoxygenation injury through enhancing autophagy via ampk-mtor signaling pathway. Eur. J. Pharmacol..

[44] Liu J., Yao Q., Xiao L., Ma W., Li F., Lai B., Wang N. (2020). Ppargamma induces nedd4 gene expression to promote autophagy and insulin action. FEBS J..

[45] Seok S., Fu T., Choi S.E., Li Y., Zhu R., Kumar S., Sun X., Yoon G., Kang Y., Zhong W. (2014). Transcriptional regulation of autophagy by an fxr-creb axis. Nature.

[46] Rui L. (2014). Energy metabolism in the liver. Compr. Physiol..

[47] Kim K.H., Moore D.D. (2017). Regulation of liver energy balance by the nuclear receptors farnesoid x receptor and peroxisome proliferator activated receptor alpha. Dig. Dis. (Basel, Switzerland).

[48] Ghosh A., Jana M., Modi K., Gonzalez F.J., Sims K.B., Berry-Kravis E., Pahan K. (2015). Activation of peroxisome proliferator-activated receptor alpha induces lysosomal biogenesis in brain cells: Implications for lysosomal storage disorders. J. Biol. Chem..

[49] Settembre C., Ballabio A. (2014). Lysosome: Regulator of lipid degradation pathways. Trends Cell Biol..

[50] Burns K.A., Vanden Heuvel J.P. (2007). Modulation of ppar activity via phosphorylation. Biochim. Biophys. Acta.

[51] Napolitano G., Esposito A., Choi H., Matarese M., Benedetti V., Di Malta C., Monfregola J., Medina D.L., Lippincott-Schwartz J., Ballabio A. (2018). Mtor-dependent phosphorylation controls tfeb nuclear export. Nat. Commun..

[52] Ballabio A., Bonifacino J.S. (2020). Lysosomes as dynamic regulators of cell and organismal homeostasis. Nat. Rev. Mol. Cell Biol..

[53] Settembre C., Zoncu R., Medina D.L., Vetrini F., Erdin S., Erdin S., Huynh T., Ferron M., Karsenty G., Vellard M.C. (2012). A lysosome-to-nucleus signalling mechanism senses and regulates the lysosome via mtor and tfeb. EMBO J..

[54] Settembre C., De Cegli R., Mansueto G., Saha P.K., Vetrini F., Visvikis O., Huynh T., Carissimo A., Palmer D., Klisch T.J. (2013). Tfeb controls cellular lipid metabolism through a starvation-induced autoregulatory loop. Nat. Cell Biol..

[55] Inpanathan S., Botelho R.J. (2019). The lysosome signaling platform: Adapting with the times. Front. Cell Dev. Biol..

[56] Islam S.M.T., Won J., Khan M., Chavin K.D., Singh I. (2019). Peroxisomal footprint in the pathogenesis of nonalcoholic steatohepatitis. Ann. Hepatol..

[57] Lodhi I.J., Semenkovich C.F. (2014). Peroxisomes: A nexus for lipid metabolism and cellular signaling. Cell Metab..

[58] Waterham H.R., Ferdinandusse S., Wanders R.J. (2016). Human disorders of peroxisome metabolism and biogenesis. Biochim. Biophys. Acta.

[59] Iershov A., Nemazanyy I., Alkhoury C., Girard M., Barth E., Cagnard N., Montagner A., Chretien D., Rugarli E.I., Guillou H. (2019). The class 3 pi3k coordinates autophagy and mitochondrial lipid catabolism by controlling nuclear receptor pparalpha. Nat. Commun..

[60] Sinha R.A., Singh B.K., Zhou J., Xie S., Farah B.L., Lesmana R., Ohba K., Tripathi M., Ghosh S., Hollenberg A.N. (2017). Loss of ulk1 increases rps6kb1-ncor1 repression of nr1h/lxr-mediated scd1 transcription and augments lipotoxicity in hepatic cells. Autophagy.

[61] Kim K., Pyo S., Um S.H. (2012). S6 kinase 2 deficiency enhances ketone body production and increases peroxisome proliferator-activated receptor alpha activity in the liver. Hepatology.

[62] Younossi Z.M., Marchesini G., Pinto-Cortez H., Petta S. (2019). Epidemiology of nonalcoholic fatty liver disease and nonalcoholic steatohepatitis: Implications for liver transplantation. Transplantation.

[63] Raza S., Rajak S., Anjum B., Sinha R.A. (2019). Molecular links between non-alcoholic fatty liver disease and hepatocellular carcinoma. Hepatoma Res..

[64] Boeckmans J., Natale A., Rombaut M., Buyl K., Rogiers V., De Kock J., Vanhaecke T., Rodrigues M.R. (2019). Anti-nash drug development hitches a lift on ppar agonism. Cells.

[65] Sanyal A.J. (2019). Past, present and future perspectives in nonalcoholic fatty liver disease. Nat. Rev. Gastroenterol. Hepatol..

[66] Patsouris D., Reddy J.K., Muller M., Kersten S. (2006). Peroxisome proliferator-activated receptor alpha mediates the effects of high-fat diet on hepatic gene expression. Endocrinology.

[67] Souza-Mello V., Gregorio B.M., Cardoso-de-Lemos F.S., de Carvalho L., Aguila M.B., Mandarim-de-Lacerda C.A. (2010). Comparative effects of telmisartan, sitagliptin and metformin alone or in combination on obesity, insulin resistance, and liver and pancreas remodelling in c57bl/6 mice fed on a very high-fat diet. Clin. Sci. (Lond.).

[68] Francque S., Verrijken A., Caron S., Prawitt J., Paumelle R., Derudas B., Lefebvre P., Taskinen M.R., Van Hul W., Mertens I. (2015). Pparalpha gene expression correlates with severity and histological treatment response in patients with non-alcoholic steatohepatitis. J. Hepatol..

[69] Abdelmegeed M.A., Yoo S.H., Henderson L.E., Gonzalez F.J., Woodcroft K.J., Song B.J. (2011). Pparalpha expression protects male mice from high fat-induced nonalcoholic fatty liver. J. Nutr..

[70] Ip E., Farrell G.C., Robertson G., Hall P., Kirsch R., Leclercq I. (2003). Central role of pparalpha-dependent hepatic lipid turnover in dietary steatohepatitis in mice. Hepatology.

[71] Yavarow Z.A., Kang H.R., Waskowicz L.R., Bay B.H., Young S.P., Yen P.M., Koeberl D.D. (2020). Fenofibrate rapidly decreases hepatic lipid and glycogen storage in neonatal mice with glycogen storage disease type ia. Hum. Mol. Genet..

[72] Waskowicz L.R., Zhou J., Landau D.J., Brooks E.D., Lim A., Yavarow Z.A., Kudo T., Zhang H., Wu Y., Grant S. (2019). Bezafibrate induces autophagy and improves hepatic lipid metabolism in glycogen storage disease type Ia. Hum. Mol. Genet..

[73] Fernandez-Miranda C., Perez-Carreras M., Colina F., Lopez-Alonso G., Vargas C., Solis-Herruzo J.A. (2008). A pilot trial of fenofibrate for the treatment of non-alcoholic fatty liver disease. Dig. Liver Dis..

[74] Laurin J., Lindor K.D., Crippin J.S., Gossard A., Gores G.J., Ludwig J., Rakela J., McGill D.B. (1996). Ursodeoxycholic acid or clofibrate in the treatment of non-alcohol-induced steatohepatitis: A pilot study. Hepatology.

[75] Basaranoglu M., Acbay O., Sonsuz A. (1999). A controlled trial of gemfibrozil in the treatment of patients with nonalcoholic steatohepatitis. J. Hepatol..

[76] Ishibashi S., Arai H., Yokote K., Araki E., Suganami H., Yamashita S., Group K.S. (2018). Efficacy and safety of pemafibrate (k-877), a selective peroxisome proliferator-activated receptor alpha modulator, in patients with dyslipidemia: Results from a 24-week, randomized, double blind, active-controlled, phase 3 trial. J. Clin. Lipidol..

[77] Ratziu V., Harrison S.A., Francque S., Bedossa P., Lehert P., Serfaty L., Romero-Gomez M., Boursier J., Abdelmalek M., Caldwell S. (2016). Elafibranor, an agonist of the peroxisome proliferator-activated receptor-alpha and -delta, induces resolution of nonalcoholic steatohepatitis without fibrosis worsening. Gastroenterology.

[78] Gonzalez-Rodriguez A., Mayoral R., Agra N., Valdecantos M.P., Pardo V., Miquilena-Colina M.E., Vargas-Castrillon J., Lo Iacono O., Corazzari M., Fimia G.M. (2014). Impaired autophagic flux is associated with increased endoplasmic reticulum stress during the development of nafld. Cell Death Dis..

[79] Tanaka S., Hikita H., Tatsumi T., Sakamori R., Nozaki Y., Sakane S., Shiode Y., Nakabori T., Saito Y., Hiramatsu N. (2016). Rubicon inhibits autophagy and accelerates hepatocyte apoptosis and lipid accumulation in nonalcoholic fatty liver disease in mice. Hepatology.

[80] Koga H., Kaushik S., Cuervo A.M. (2010). Altered lipid content inhibits autophagic vesicular fusion. FASEB J..

[81] Park H.W., Park H., Semple I.A., Jang I., Ro S.H., Kim M., Cazares V.A., Stuenkel E.L., Kim J.J., Kim J.S. (2014). Pharmacological correction of obesity-induced autophagy arrest using calcium channel blockers. Nat. Commun..

[82] Rodriguez-Navarro J.A., Kaushik S., Koga H., Dall’Armi C., Shui G., Wenk M.R., Di Paolo G., Cuervo A.M. (2012). Inhibitory effect of dietary lipids on chaperone-mediated autophagy. Proc. Natl. Acad. Sci. USA.

[83] Inami Y., Yamashina S., Izumi K., Ueno T., Tanida I., Ikejima K., Watanabe S. (2011). Hepatic steatosis inhibits autophagic proteolysis via impairment of autophagosomal acidification and cathepsin expression. Biochem. Biophys. Res. Commun..

[84] Nakadera E., Yamashina S., Izumi K., Inami Y., Sato T., Fukushima H., Kon K., Ikejima K., Ueno T., Watanabe S. (2016). Inhibition of mtor improves the impairment of acidification in autophagic vesicles caused by hepatic steatosis. Biochem. Biophys. Res. Commun..

[85] Fukuo Y., Yamashina S., Sonoue H., Arakawa A., Nakadera E., Aoyama T., Uchiyama A., Kon K., Ikejima K., Watanabe S. (2014). Abnormality of autophagic function and cathepsin expression in the liver from patients with non-alcoholic fatty liver disease. Hepatol. Res..

[86] Allaire M., Rautou P.E., Codogno P., Lotersztajn S. (2019). Autophagy in liver diseases: Time for translation?. J. Hepatol..

[87] Upadhyay A., Anjum B., Godbole N.M., Rajak S., Shukla P., Tiwari S., Sinha R.A., Godbole M.M. (2019). Time-restricted feeding reduces high-fat diet associated placental inflammation and limits adverse effects on fetal organ development. Biochem. Biophys. Res. Commun..

[88] Morel E., Mehrpour M., Botti J., Dupont N., Hamai A., Nascimbeni A.C., Codogno P. (2017). Autophagy: A druggable process. Annu. Rev. Pharmacol. Toxicol..

[89] Cai H., Qin Y.L., Shi Z.Y., Chen J.H., Zeng M.J., Zhou W., Chen R.Q., Chen Z.Y. (2019). Effects of alternate-day fasting on body weight and dyslipidaemia in patients with non-alcoholic fatty liver disease: A randomised controlled trial. BMC Gastroenterol..

[90] Zhou J., Farah B.L., Sinha R.A., Wu Y., Singh B.K., Bay B.H., Yang C.S., Yen P.M. (2014). Epigallocatechin-3-gallate (egcg), a green tea polyphenol, stimulates hepatic autophagy and lipid clearance. PLoS ONE.

[91] DeBosch B.J., Heitmeier M.R., Mayer A.L., Higgins C.B., Crowley J.R., Kraft T.E., Chi M., Newberry E.P., Chen Z., Finck B.N. (2016). Trehalose inhibits solute carrier 2a (slc2a) proteins to induce autophagy and prevent hepatic steatosis. Sci. Signal..

[92] Lim H., Lim Y.M., Kim K.H., Jeon Y.E., Park K., Kim J., Hwang H.Y., Lee D.J., Pagire H., Kwon H.J. (2018). A novel autophagy enhancer as a therapeutic agent against metabolic syndrome and diabetes. Nat. Commun..

[93] Kim S.H., Kim G., Han D.H., Lee M., Kim I., Kim B., Kim K.H., Song Y.M., Yoo J.E., Wang H.J. (2017). Ezetimibe ameliorates steatohepatitis via amp activated protein kinase-tfeb-mediated activation of autophagy and nlrp3 inflammasome inhibition. Autophagy.

[94] Nakade Y., Murotani K., Inoue T., Kobayashi Y., Yamamoto T., Ishii N., Ohashi T., Ito K., Fukuzawa Y., Yoneda M. (2017). Ezetimibe for the treatment of non-alcoholic fatty liver disease: A meta-analysis. Hepatol. Res..

[95] Estes C., Razavi H., Loomba R., Younossi Z., Sanyal A.J. (2018). Modeling the epidemic of nonalcoholic fatty liver disease demonstrates an exponential increase in burden of disease. Hepatology.

[96] Erol A. (2007). The functions of ppars in aging and longevity. PPAR Res..

[97] Rubinsztein D.C., Marino G., Kroemer G. (2011). Autophagy and aging. Cell.

